# A Review of the Efficacy of Nanomaterial-Based Natural Photosensitizers to Overcome Multidrug Resistance in Cancer

**DOI:** 10.3390/pharmaceutics16091120

**Published:** 2024-08-24

**Authors:** Jagadeesh Rajaram, Lokesh Kumar Mende, Yaswanth Kuthati

**Affiliations:** 1 Department of Biochemistry and Molecular Medicine, National Dong Hwa University, Hualien 974, Taiwan; jagadeesh31994@gmail.com; 2 Department of Anesthesiology, Cathy General Hospital, Taipei 106, Taiwan; lokeshyv66@gmail.com

**Keywords:** photodynamic therapy, nanotechnology, multidrug resistance, natural PSs

## Abstract

Natural photosensitizers (PS) are compounds derived from nature, with photodynamic properties. Natural PSs have a similar action to that of commercial PSs, where cancer cell death occurs by necrosis, apoptosis, and autophagy through ROS generation. Natural PSs have garnered great interest over the last few decades because of their high biocompatibility and good photoactivity. Specific wavelengths could cause phytochemicals to produce harmful ROS for photodynamic therapy (PDT). However, natural PSs have some shortcomings, such as reduced solubility and lower uptake, making them less appropriate for PDT. Nanotechnology offers an opportunity to develop suitable carriers for various natural PSs for PDT applications. Various nanoparticles have been developed to improve the outcome with enhanced solubility, optical adsorption, and tumor targeting. Multidrug resistance (MDR) is a phenomenon in which tumor cells develop resistance to a wide range of structurally and functionally unrelated drugs. Over the last decade, several researchers have extensively studied the effect of natural PS-based photodynamic treatment (PDT) on MDR cells. Though the outcomes of clinical trials for natural PSs were inconclusive, significant advancement is still required before PSs can be used as a PDT agent for treating MDR tumors. This review addresses the increasing literature on MDR tumor progression and the efficacy of PDT, emphasizing the importance of developing new nano-based natural PSs in the fight against MDR that have the required features for an MDR tumor photosensitizing regimen.

## 1. Introduction

Cancer is the second largest cause of death worldwide with one out of every four deaths resulting from cancer [1]. Cancer is caused by mutations in the DNA. In the United States alone, 611,720 deaths are estimated to be from cancer in 2024 [2]. Cancer develops resistance to standard chemotherapy and radiation, thereby reducing the survival rate [3]. Chemotherapy and other currently available therapies are known to be very effective in treating cancer in the initial stages. However, these therapies also kill the normal healthy cells along with the tumor cells during treatment, resulting in adverse effects on the patient’s health. As a result, developing targeted cancer therapeutics, which specifically destroy cancer cells, is the key to enhancing the therapeutic outcome, with the minimization of side effects [4,5,6].

In recent years, PDT has provided patients with the possibility of receiving adequate treatment. PDT has the advantages of good selectivity, less severity, low toxic effects and possible complications, and repeatability as a noninvasive cancer therapeutic [7,8]. PSs are substances that, when activated by light, yield toxic ROS that have cytotoxic impacts on cancer. Numerous PSs for chemotherapy were explored during this process [9]. The majority of the existing PSs used in therapies have drawbacks such as toxicity, which limit their clinical application [10]. Natural PSs have recently garnered significant attention due to their peculiar properties. In particular, porphyrin and its derivatives have gained substantial interest due to their low cytotoxicity and good in vivo biocompatibility making natural products, a good alternative to traditional PSs [11]. Over the last few decades, several natural extract-based PSs have been developed. In particular, curcumin, berberine, pheophytin, 5-aminolevulinic acid, porphyrin, hypocrellin, bacteriochlorin, and pheophorbide are some of the naturally occurring PSs with potential clinical applications. Recently, a third-generation-based PS was developed using porphyrin-based hematoporphyrin, which combines the efficiency of previous generations to generate a highly efficient PDT regime. The lack of stability and a weak adsorption band in the phototherapeutic window make natural PSs unsuitable for their usage in PDT for clinical applications. Various nanoparticles, including organic nanoparticles, inorganic nanoparticles, and carbon nanoparticles, were developed to overcome the limitations associated with natural PSs [12,13]. Nanoparticles have several advantages, such as controlled size and morphology for distinct demands, modification with specific functional groups for targeted delivery, and an EPR effect that can enhance the retention of nanoparticles in tumor sites, resulting in enhanced PDT efficiency [14,15,16]. 

MDR development is one of the major challenges associated with traditional chemotherapeutic agents. The combination of natural PSs with nanoparticles has great potential in overcoming MDR [17,18]. This review will summarize the different natural PSs that have been proposed for PDT applications by incorporation into nanoparticles and their efficacy in treating MDR, as well as the impact of nanoparticle incorporation on the chemical and physical properties of PSs.

### 1.1. MDR in Cancers

**Figure 1:**
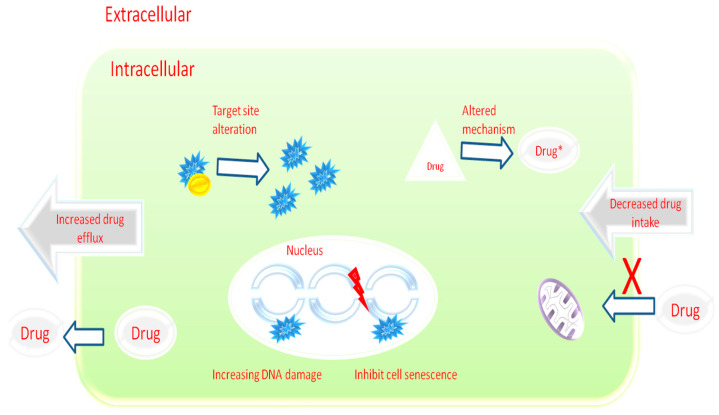
Schematic illustration of mechanisms contributing to the development of MDR in cancer cells. Cancer cells have developed a wide range of mechanisms to fight against various therapeutical drugs. Abc transporters, alterations in the apoptosis pathway, drug inactivation through cellular metabolism, and mutations in cellular and drug targets, an enhanced DNA repair mechanism, are well-known mechanisms. Altered mechanism in the cancer cell nullifies the effect of drug by changes in various properties which denoted as drug*.

MDR limits the efficacy of most of the chemotherapeutic agents in treating cancer. The phenomenal changes in the cancer cell microenvironment render chemotherapeutic agents ineffective. Changes at the cellular level reduce the potency of similar structured chemotherapeutic agents, resulting in cancer recurrence [19,20,21]. MDR is gained through a variety of intrinsic and extrinsic mechanisms to reduce the therapeutic efficacy of cancer drugs [22,23]. This section will explain the mechanisms of MDR at various levels of the cellular microenvironment, as represented in Figure 1. Intrinsic resistance is a method of evading treatment that is developed within cancer cells. When a cell is treated against drug, an extrinsic pathway is formed in order to expel therapeutic drug. The ATP-binding cassette (ABC) pumps out therapeutic drugs or can cause oncogene mutation, escaping cell death. ABC transporters are two nucleotide-binding domains classified as ABC transporters (A-G) [24]. Different ABC transporters serve different functions and are found on the cell membrane’s surface [25]. The ABC transporters’ primary functions are to remove the substrate from the cell using ATP energy. ABC transporters can pumps out chemotherapeutic drugs outside the cancer cell with the help of ATP [25]. According to various reports, ABCB1 (P-glycoproteins (P-gps)), ABCG2, and ABCC1 overexpression may play a role in MDR [26,27]. P-gp inhibition can promote apoptosis in cisplatin-resistant human colon cancer HCT116/DDP cells [24,28]. The tumor environment’s antioxidant capacity causes chemo-resistance. Decreased reactive oxygen species (ROS) production and an oscillation in glutathione enzyme (GSH) levels can lessen oxidative stress and promote MDR. The endoplasmic reticulum (ER) also functions in the MDR to prevent apoptosis by lowering the concentration of unfolded proteins in the cell [29]. In MDR cancer, increasing ER calcium storage shows an increase in ER stress and increases P-gp efflux activity [30,31]. Some studies recovered calcium entry in the endoplasmic reticulum (ER) and elevated ER stress. The DOX level in the cancer cells was raised by a large amount by reducing the efflux activity of P-glycoprotein which has proven to have a crucial effect in tackling resistance by triggering apoptosis pathways and overcoming DOX resistance in MCF-1/KCR cell lines [32]. Hypoxia conditions impact MDR development in cancers through the upregulation of hypoxia-inducible factors (HIFs) 1a and 2a [33]. Changes in the oxygen levels in the cancer environment will make the cell function with a low level of oxygen. The cancer cell requires a high level of oxygen to function. Various proteins such as hypoxia-inducible factors 1a and 2a required lot of oxygen in order to survive [34]. Breast cancer MCF-7 cells are resistant to doxorubicin under hypoxic conditions [35]. The downregulation of DRP1 may suppress mitochondria fusion and results in mitophagy led to deployment of cisplatin resistance in human ovarian cancer cells [36]. The failure of down regulation of such genes results in resistance and stemness via DRP1 induced mitochondria fragmentation with enhanced glycolysis in ovarian cancer cells.

Stem cells are a self-renewable subpopulation of cells that develop tissue for various organs. These subsets of cells are the replacements for damaged organs. Thus, these adult stem cells play a huge role in repairing tissues. Even cancers have a self-renewable subpopulation of cells through differentiation. These stem cells possess characteristics of both stem cells and cancer cells. Stem cells receive signals from immune cells, and cancer-associated fibroblasts present inside the tumor environment. This is one of the key reasons for the failure of cancer treatment through chemo-resistance [37,38,39].

Resistance development by cancer cells is also associated with the epithelial-to-mesenchymal transition (EMT). Epithelial cells undergo a reversal to gain migration and invasion properties akin to those of mesenchymal cells during processes such as wound healing, immune evasion, and metastasis. Non-cell instructive signals from the tumor microenvironment play a crucial role in cell survival. The EMT controls tumor initiation, progression, metastasis, and resistance to various types of anticancer therapies. Still, the mechanism behind the EMT-induced resistance to therapy in tumors is unclear [40,41].

Additional enzymes, such as the signal transducer and the activator of the transcription (STAT) protein, are increased by the resistant cells to prevent cell death caused by various drugs. An amino acid domain (NH_2_), a coiled-coil domain (CCD), a DNA-binding domain (DBD), a linker domain, an SRC homology 2 (SH2) domain for phosphorylation and dimerization, and a C-terminal transactivation domain (TAD) are the functionalities present in the protein responsible for the resistance mechanism. Stat protein family 1–6 intracellular transcription factors handle differentiation, proliferation, and apoptosis. They receive signals from the surface receptors and transmit signals to the nucleus whenever the cell needs to undergo modification. The overexpression of protein in the cancer environment causes cancer initiation, progression, metastasis, resistance to chemotherapy, and poor survival outcomes. Servian, c-Myc, cyclin D1, B-cell lymphoma-2 (Bcl-2), and B-cell lymphoma-extra-large (Bcl-xL) are the identified genes usually overexpressed and linked to the stat proteins for their role in the resistance mechanisms. The activation of signaling pathways related to the STAT protein handles cancer cell proliferation, antiapoptosis, etc. [42,43,44,45,46,47].

The nuclear phosphoprotein p53 is a tumor suppressor gene that functions as a cell cycle checkpoint in the repair of damaged DNA and in cell growth control by acting as a transcription factor [48]. An alteration in the p53 gene handles the resistance in the cancer cells to evade apoptosis. Nuclear factor kappa-light-chain-enhancer of activated B cells (NF-κB) is also responsible for the resistance gained by the cancer cells, by binding with DNA to undergo transcription, cytokine production, and cell survival [49]. 

### 1.2. Photodynamic Therapy

Thermal, ultrasound, and photodynamic therapies are the three significant categories used in cancer treatment. Various combinations of these therapies for cancer have been proven to enhance therapeutic efficacy in comparison with individual therapy. Among these, photodynamic therapy is a non-conventional strategy that can induce cancer cell death through reactive oxygen species [50]. During ancient times in India, Egypt, and China, sunlight-based therapy was used to address various skin abnormalities. Over time, the treatment has reached minimal side effects because of the improvement in the technology. Many studies have been conducted to explore alternative treatment methods using light-based approaches. In 1903, the Nobel Prize acknowledged Niels Finsen for treating cutaneous tuberculosis using phototherapy with the help of ultraviolet rays. Following this, J. Prime used eosin sensitization through sunlight to treat epilepsy. Later, H. Tappeiner and A. Jesionek used eosin to treat skin tumors with the help of white light. H. Tappeiner and A. Jodlbauer coined the term “photodynamic action” when they discovered the oxygen requirement in the photosensitization process [51,52]. The first successful removal of a tumor from an animal species paved the way for the development of a new era for this technique.

**Figure 2:**
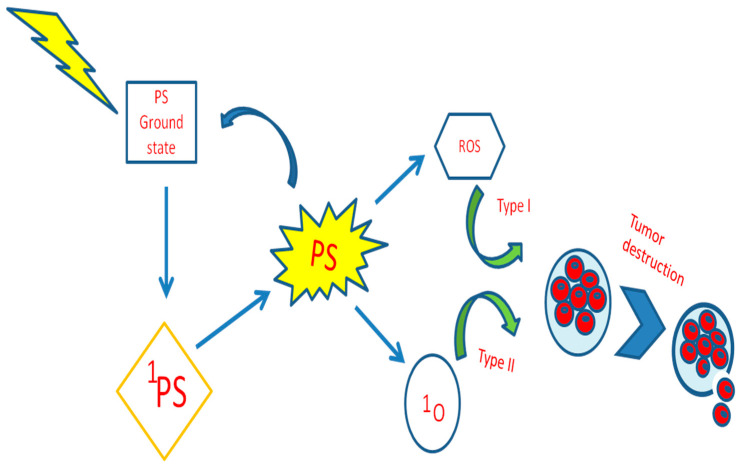
Schematic illustration of the molecular mechanism of PDT. The Jablonski diagram illustrates the transitions of PSs from the ground state to the excited state, both for type I and type II reactions. This transition occurs when the PSs absorb light of a particular wavelength, resulting in the generation of free radicals that can cause cellular damage.

Photofrin was the first approved PDT-based drug molecule for bladder cancer therapy in 1993 [53]. Currently, many commercial PSs are available on the market to treat cancer. Photodynamic therapy relies on three main components: a PS, light, and molecular oxygen. The PS is in an inactive state when it enters the cell. It can be activated with an appropriate light source. Oxygen is the crucial component required for the therapy to be effective. Once activated, the PS can interact with the surrounding oxygen and undergo two types of reactions, as represented in Figure 2. The PS absorbs the light, excites it to a higher state in its electron configuration, and produces free radicals that are toxic to the cell. These radicals interact with targeted molecules and affect their function, which eventually leads to cell death. In contrast, singlet oxygen occurs when triplet-state molecules interact with oxygen present inside the cell. They are toxic and highly reactive. The formation of singlet oxygen relies on the process of triplet–triplet annihilation. Both types of ROS reactions take place at the same time. To achieve effective treatment, it is crucial to maintain a harmonious equilibrium between the concentration of the PS, oxygen molecules, and the affinity of the PS with the substrate. ROSROS and singlet oxygen are highly reactive and have a shorter half-life. Therefore, when localizing the PS within the intended system, these factors should be considered [54].

### 1.3. Role of PS in Resistance Cell Death

Phototherapy induces cell death through three common modes: necrosis, autophagy, and apoptosis. PSs are specifically targeted and localized within the cancer cell, affecting each intercellular component [55]. PSs trigger two separate pathways of cell death. The first pathway involves the degradation of lysosomes caused by photodamage, known as lyse-PDT. The second pathway occurs when the mitochondria are damaged by light, referred to as mito-PDT. The leakage of proteases into the cytosol leads to the cleavage and activation of the proapoptotic factor tBID within the lysosome [56,57]. Within the mitochondria, PSs selectively trigger the activation of the proapoptotic protein BAX through the impairment of the antiapoptotic protein [58]. The antiapoptotic proteins remain intact within the cytosol after being separated from the outer membrane of the mitochondria. The release of cytochrome c triggers the activation of effector caspase, which is accountable for the irreversible apoptosis of cancer cells. An imbalance in proapoptosis is observed during chemotherapy, resulting in the development of resistance. The overexpression of antiapoptotic BCL-2 family proteins, such as BCL-2 and BCL-XL, primarily causes the evasion of these pathways. These mechanisms are observed in drug resistance. Another class of proteins responsible for resistance includes ATP-binding cassette transporter proteins like ABCC1, ABCB1, and ABCG2. These proteins typically expel unwanted molecules from the cell, including the efflux of anticancer drugs and sequestration [59]. 

### 1.4. Limitations of Photodynamic Therapy

Cancer cells can develop resistance to PDT, similar to chemotherapy. Drug efflux proteins responsible for chemotherapy resistance can also cause resistance to PDT by the efflux of PSs. Multidrug resistance phenotype P-glycoprotein and ATP-binding cassettes are the key mediators of drug resistance acquirement [60,61,62,63].

Heat-shock proteins (HSPs) handle stress management and play an important role in thermal stress and homeostasis. HSPs have been reported to have a protective effect against PDT in cancer cells. ROS, the byproduct of PDT, can target specific cell organelles, leading to cell death through apoptosis, necrosis, and autophagy pathways. Autophagy involves the removal of cell debris from normal cells for cell survival and proliferation. Some reports confirm that autophagy can play a cytoprotective role against PDT-induced damage in cancer cells [64].

Hypoxia-inducing factor-1 alpha is another key mechanism that nullifies PDT. PDT induces hypoxia because of oxygen consumption and vascular damage [65,66]. Cancer cells change the mitochondrial size in the process of developing resistance against PDT, reducing the uptake and localization of PSs. The physical and chemical properties of the PSs need to be studied well before their usage for PDT applications [67]. Nanotechnology addresses some key challenges faced in the clinical and scientific advancement in PDT for cancer PDT. The entrapment of natural PSs into nanoparticles has several benefits over free PSs. A tunable size, shape, and surface charge and the ease of surface functionalization with desired moieties are some important characteristics that make NPs ideal for cancer PDT treatment. The correct localization of PSs and the maintenance of desired intracellular concentrations of PSs are some of the important factors essential for desired PDT outcomes. Long exposure times and a high PS concentration would also affect the normal cells, apart from the cancer cells. So, these parameters should be addressed for successful PDT. Recently, PS-loaded nanoparticles for MDR cancer have gained significant attention. Because of the multiple challenges associated with treating MDR, there is a need for modern strategies that can counteract all the current limitations associated with traditional PDT. Functionalizing nanoparticles with ABC transporter inhibitors is one of the most commonly used strategies for counteracting MDR [68,69,70]. However, it has some limitations, such as nonspecific toxicity and undesirable pharmacokinetic interactions, and also challenges in formulation, such as solubility, permeability, and stability. Nanoparticles make it possible to co-deliver natural PSs in combination with chemotherapeutic drugs, which is an ideal strategy for overcoming MDR. Several aspects of natural PSs need to be studied carefully for their clinical translation [68,69,70].

## 2. Naturally Occurring PSs

Since ancient times, natural components have been used in the medicinal field as antimicrobial agents. Various diseases have been treated with natural extracts. In this section, we mainly focus on the benefits of natural PSs for PDT. Natural compounds are abundant in fruits, vegetables, plants, fungi, bacteria, etc., all of which contain compounds with phototherapeutic properties. An unprecedented revitalization of natural PSs has been taking place in the medicinal field in recent years. Modern advances in analytical chemistry help in identifying pharmacologically active compounds and their properties. Photosensitization is one such property within natural extracts, where a component can absorb a particular spectrum of light and produce toxic products like ROS. Many natural compounds have been screened to find out their phototoxic properties [71,72,73,74]. Clinically approved natural photosensitizers were first developed in the 1970s by Dr. T. Dougherty and his collaborators. Hematoporphyrin (Hp)-based Photofrin ^®^ was a first-generation PS developed with some limitations, including chemical purity, tissue penetration that leads to skin hypersensitivity, photo bleaching, and low absorption, which reduced its efficiency. These factors led to the development of second-generation-based NPs for improved PDT. Second-generation PSs also had some limitations, such as dark toxicity, which was the major concern in PDT. These factors demand the development of PSs with improved affinity towards tumor tissue without affecting the healthy surrounding cells [75].

Also, different types of nanoparticle such as inorganic, organic, and hybrid nanoparticles have played a huge role in improving PDT efficacy [75]. Table 1. lists the different types of nanoparticles used for delivering various natural PSs for PDT against cancer. This review mainly focused on the various common natural PSs loaded with various nanoparticles and their phototoxic properties against cancer summarized below. Table 2. lists the various natural PSs with their wavelength and structures. This review mainly focused on the various common natural PSs loaded with various nanoparticles and their phototoxic properties against cancer summarized below.

### 2.1. Curcumin

Curcumin is a phytochemical extracted from *Curcuma longa* L. (turmeric). It is available in ketone form and enol forms. Curcumin has an anticancer effect by inhibiting tumor cell proliferation. Because of this, many researchers use it as a therapeutic agent against various microorganisms and cancer cells. Curcuminoids can absorb blue light in the spectrum of light and produce ROS to inhibit cancer cells. It is used as a PS in photodynamic therapy against various cancer cell lines. Curcumin shows good toxicity against cancer cells without harming normal cells [134,135,136,137,138,139].

Its hydrophobic nature is an important limitation in its usage for anticancer applications. The low oral availability, rapid metabolism, and low cellular uptake are also a major concern for curcumin in the application of PDT. Hence, there is a need to tune the physicochemical properties for biomedical applications. Various factors, such as increasing the bioavailability and overcoming the rapid metabolism of curcumin in vivo, are the key factors one should consider before developing curcumin-based PSs. Hence, there is a need to tune the physicochemical properties for biomedical applications. Various nanoparticles, such as magnetic nanoparticles, gold nanoparticles, mesoporous silica nanoparticles, layered double hydroxides, MoS2 nanoparticles, quantum dots, graphene, and curcumin–metal complexes, were used to improve the solubility and photodynamic properties of curcumin [140,141,142,143,144].

The response of cancer to curcumin nanoparticles may vary because of the intricate environment, diverse survival pathways, abnormal growth rates, and other factors. However, enhancing their effectiveness is possible by functionalizing different targeting moieties for specific cancer cells. Combining them with other photodynamic therapy agents or chemotherapeutic drugs often yields superior results [76,145].

Curcumin nanoparticles loaded with layered double hydroxide were synthesized and used in the treatment of the MDA-MB 231 breast cancer cell line [77]. The efficacy of curcumin PDT is superior to that of free curcumin at a concentration of 100 µg/mL. The production of reactive oxygen species (ROS) appears to be significantly elevated, leading to the induction of apoptosis in breast cancer cells compared with free curcumin. The same research group observed a similar effect of methylene blue–curcumin PDT against the mad-mb 231 cell line [107]. The complex shows superior photodynamic efficiency compared to the salicylate–methylene blue complex. Additionally, mda-mb 231 cells were subjected to treatment with PLGA nanoparticles containing curcumin to combat metastasis [108]. A cell survival rate of 3.59% was noted after a 30 min irradiation. The PDT treatment changed the cell structure involving curcumin-loaded PLGA nanoparticles. A Ga–curcumin complex was formed using a 1,10-phenanthroline chelator. Breast cancer cell survival decreased with an increase in the complex’s dosage. ROS production was consistent across all metal complexes [146].

The combination therapy involved the utilization of a curcumin–gold nanocomposite to facilitate photothermal and photodynamic therapy [78,79,81]. An ROS-responsive nanocomposite was synthesized with upconversion nanoparticles to be used in the treatment of melanoma cell lines. The successful eradication of melanoma was achieved through the synergistic effects of photothermal therapy (PTT) and photodynamic therapy (PDT), leading to apoptosis and ferroptosis. The curcumin–silica nanocomplex exhibited significant efficacy against melanoma cancer cells by inducing the production of reactive oxygen species (ROS) [83]. The results confirmed that cell death was achieved through ROS. ICG&Cur@MoS2 nanoparticles were used for synergetic PTT-PDT against hepatocellular carcinoma [147].

**Figure 3:**
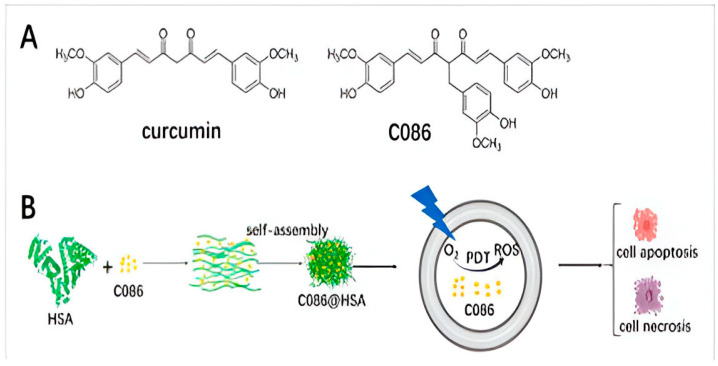
(**A**) Schematic illustrations of the chemical structure of curcumin and its derivative C086 and the preparation of C086@Hsa nanoparticles and the process of photodynamic therapy. C086, a derivative of curcumin, was loaded into the albumin-based nanoparticles by a simple self-assembly method. (**B**) The formation of self assembled curcumin derivative loaded Human serum albumin for PDT against cancer cells “Reprinted with permission from Ref. [147]. 2021, Elsevier B.V.”.

Albumin nanoparticles were loaded with curcumin derivative C086, a novel heat shock protein inhibitor (Figure 3A) [148]. Hela cells exhibited cell cycle arrest upon exposure to blue light for 10 min. The curcumin nanodrug, devoid of any carriers, demonstrated superior toxicity compared to free curcumin [114]. The nanoparticles stimulated the formation of ROS through the MAPK pathway in 4t1 cells in contrast to free curcumin (refer to Figure 3B). Cancer cell lines, including a human gastric cancer cell line (MKN45) and a human gastric epithelial mucosa (non-cancer) cell line (GES), were treated with curcumin-encapsulated chitosan/tpp nanoparticles [149]. ROS production was observed against a cancer cell line without significant adverse effects. Pancreatic carcinoma was treated with chlorine e6–curcumin conjugates [102]. The findings from the nanoconjugates showed that the Ic50 values fell within the range of 35–41 nm, demonstrating favorable phototoxicity.

Curcumin-loaded solid lipid nanoparticles (SLNs) can specifically target mitochondria, resulting in elevated expression levels of caspase 9 and caspase 3, as well as a shift in the bcl-2/bax ratio. This is achieved by disrupting the mitochondrial membrane, ultimately leading to an increase in reactive oxygen species (ROS) [114]. Blue-light-activated curcumin nanoparticles exhibited efficacy against U251 glioma, B16 melanoma, and H469 lung cancer cells [102]. In the corresponding cell lines, the observation of c-Jun N-terminal kinase, mitochondrial depolarization, caspase 3 activation, and the cleavage of poly (ADP-ribose) polymerase resulted in apoptosis. The combination of doxorubicin and curcumin nanoparticles induced an ROS-mediated p53-dependent apoptotic pathway in the HepG2 cell line [150]. Cellular cycle cessation was additionally observed in the photoactive curcumin nanoparticles within the corresponding cellular lineages. Arrest at the G2/M and S phases of the cell cycle, along with DNA fragmentation, was observed [151]. The photoactive curcumin nanoparticles exhibited the inhibition of cyclin-dependent kinases and cell-dependent proteins [84]. These discoveries contribute to the effective suppression of cancerous cells. Although the in vivo and in vitro models have shown a maximum success rate, certain limitations hinder their efficacy in clinical trials. Overcoming these limitations will pave the way for a successful approach to the fight against cancer.

### 2.2. Porphyrin

Porphyrin, a conjugated skeleton macrocyclic compound, serves as the primary constituent of hemoglobin, cytochrome, and chlorophyll. It is composed of four pyrrole subunits interconnected by four methine bridges. Given its ability to selectively accumulate at the intended tumor site and exhibit prolonged activation, porphyrin and its derivatives hold promise as a potential candidate for phototherapy [152,153,154,155]. During the 1980s and 1990s, hematoporphyrin derivatives were initially utilized as a PDT agent commercially known as photofrin. Later, photofrin received approval over many countries for treatment of various cancers. Its weak absorption in the red region, complex composition, lack of selectivity, and certain side effects hindered its clinical use. Most of the porphyrin structure causes aggregation because of its design, resulting in reduced ROS production. The primary goal is to enhance efficiency by minimizing aggregation at the intended site. Porphyrin may accumulate in healthy tissues, leading to potential damage [156,157]. To address these challenges, nanoparticles can be utilized to enhance the biocompatibility of porphyrin. Both inorganic and organic nanoparticles can serve as effective agents in achieving this goal. A wide range of nanoparticles, such as metal–organic frameworks, mesoporous silica nanoparticles, liposomes, peptides, quantum dots, albumin, and lactosomes, have been employed as carriers for porphyrin. By incorporating multimodality imaging functionalities, porphyrin can visualize tumor cells both in vivo and in vitro. Techniques such as fluorescence imaging, photoacoustic imaging, and MRI imaging can predict the outcomes of the therapy. Combining different therapies such as chemodynamic therapy, immune therapy, photothermal therapy, chemotherapy, and sonodynamic therapy can synergistically enhance the therapeutic outcomes for cancer treatment [158,159]. While there have been many reviews discussing the applications of porphyrin, this discussion focuses on the recent advancements in using multifunctional nanoparticles loaded with porphyrin for photodynamic therapy against cancer [160,161,162].

**Figure 4:**
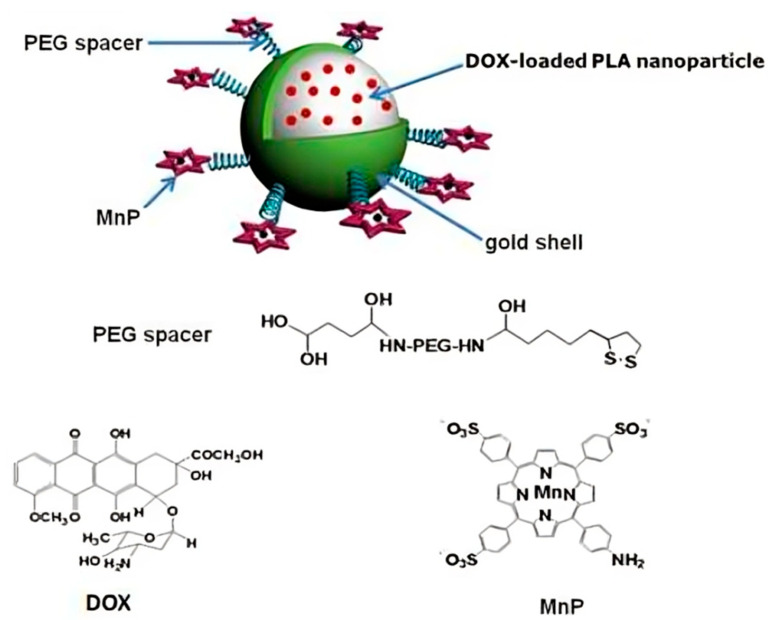
A diagrammatic representation of the synthetic process of DOX@PLA@Au−PEG−MnP NPs. Adapted with permission from Ref. [167].

A novel nanoparticle composed of zinc, porphyrin, and silica was created for the combined treatment of tumors [104]. A silica shell was utilized to successfully fabricate photo-immunotherapy, enabling the loading of immune adjuvants [128]. The immune response played a crucial role in significantly inhibiting tumor growth. Liposome-loaded porphyrins were used for targeted photo-chemotherapy in human MIA Paca-2 tumors [115]. Liposomes containing cabazitaxel demonstrated stability in the serum [129]. When compared to monotherapy, combination therapy has a significantly greater impact on tumor growth. Successful tumor ablation was observed using a chemo-phototherapy stimulator loaded with Mn (III)-TCPP MOF, leading to enhanced innate and adaptive antitumor immune responses (Figure 4) [163].

An arginine–peptide complex was constructed to enhance the porphyrin photodynamic therapy (PDT) impact on the hypoxic environment created by the tumor [164]. Arginine is known to generate nitric oxide, which specifically affects mitochondrial cellular respiration and improves the efficiency of PDT [164]. A higher concentration of nanoparticles was observed following intravenous injection in mice with tumors [164]. The laser irradiation of nanoparticles showed a reduction in tumor growth, addressing the hypoxia caused by the tumor’s resistance to PDT [164]. Chromatin decompaction results in the simultaneous activation of innate and adaptive antitumor immunity, leading to immunogenic cell death induced by PDT. The nucleus-targeted PDT system, based on self-assembly, exhibited a significant inhibition of tumor growth and metastasis when exposed to light in various xenograft tumor models. A cancer-cell-membrane-coated metal–organic framework was utilized for the combination of ferroptosis and phototherapy in the treatment of breast cancer [165]. The cancer cell membrane homology aids in the evasion of immune clearance by camouflaging the nanoparticle, allowing it to accumulate at the tumor site. By utilizing the Fenton reaction in the tumor environment, nanoparticles generate ions that induce ferroptosis, enhancing the efficiency of photodynamic therapy [166]. The synergistic therapeutic effects triggered by the tumor microenvironment contribute to the advancement of effective nanomedicine. The drug loading efficiency of poly (D, L-lactide-co-glycoside) (PLGA)- and poly (D, L-lactide) (PLA)-loaded porphyrin is significantly improved, resulting in a higher therapeutic index compared to free drugs [167]. Hence, the nonformulation offers a significant benefit in terms of specifically targeting cancer cells. The attachment of PEG to porphyrin enabled the prolonged circulation of the nonformulation [168]. The synthesized nanotherapeutic agent comprises an Au shell with PEG for the attachment of Mn–porphyrin [130]. When compared to a single agent, these nanoparticles show superior outcomes because of their combinational effect. Therefore, by functionalizing with different polymers under specific conditions, the phototoxicity of the PS can be enhanced [131].

Despite the significant advantages demonstrated by the many nanoparticles loaded with porphyrin in both in vivo and in vitro methodologies, it is important to note that they are still in the early stages of clinical trials. Several aspects need to be addressed to fully understand the potential of porphyrin-loaded nanoparticles in translational medicine. These include overcoming transport barriers, enhancing drug uptake, addressing distribution hurdles, and assessing long-term effects. However, it is worth noting that these nanoparticles hold great promise for the future of medicine.

### 2.3. Pheophorbide a (PA)

Pheophorbide a (PA) is a chlorophyll A derivative found in plants and algae. PA exhibits an ultraviolet spectrum with a Soret band at 390 nm and Q bands ranging from 500 to 700 nm. Notably, the Q band at 670 nm displays a higher intensity [169]. Several studies have shown the efficacy of PA in treating different cancer cell lines [170]. This active compound is derived from plant extracts used in Chinese medicine. Remarkably, it has been found to have no significant toxicity on healthy cells [116]. PA causes skin irritation after exposure to light. Some patients experiences allergy-like symptoms after exposure to the PA in regular intervals. Long PA exposure in mice also induced skin lesions and even death in laboratory experiments. These side efforts should be minimized when developing PA-based PSs.

In cancer research, PA has garnered increased attention in recent years. Exciting results have emerged from combining it with various chemotherapeutic drugs. Researchers have synthesized a range of nanoparticles to enhance PA’s therapeutic potential, paving the way for clinical trials.

**Figure 5:**
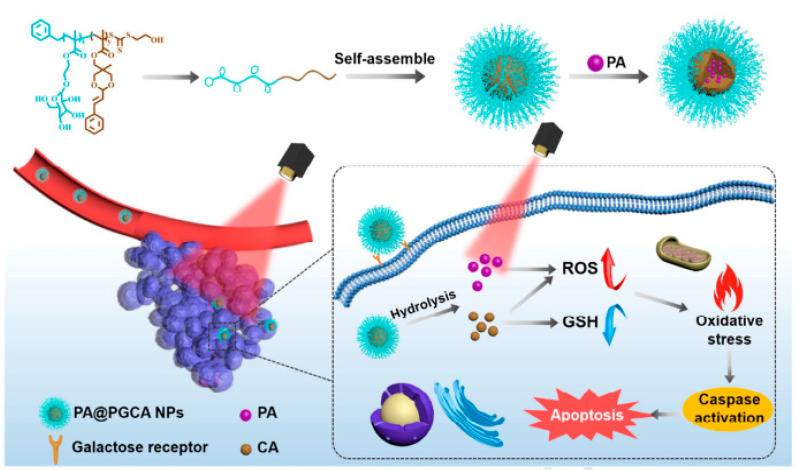
A schematic illustration of the synthesis process of PGCA@PA nanoparticles and the mechanism by which they induce cell death using light exposure. The acid-responsive polygalactose-co-polycinnamaldehyde polyprodrug was combined with cinnamaldehyde (CA) and PS pheophorbide A (PA). The galactose receptor present in cancer cells is responsible for the internal breakdown of PGCA NP and the generation of ROS levels upon exposure to light. This ultimately leads to the apoptosis of cancer cells. “Reprinted with permission from Ref. [120]. 2020. Elsevier B.V.”.

For instance, alginate nanoparticles were prepared for the combinational therapy of PA and doxorubicin [117]. PA was conjugated via a redox-sensitive linkage to an alginate nanoparticle [171]. These nanoparticles generated singlet oxygen alongside anticancer effects within B16 tumor-bearing mice [118]. The nanoparticles facilitated the induction of INF-λ in the serum of the mice [118]. Micelles were created using doxorubicin and PA for chemo-photodynamic treatment against B16 melanoma [120]. With the combination, a 73.5% inhibition of tumor growth was observed when compared to the non-irradiated nanoparticle and free drug [120]. The photodynamic therapy (PDT) effect of nano-transfersomes loaded with PA against HELA and A549 cells was clear. Notably, the lecithin-based nano-transfersomes exhibited enhanced phototoxicity with no signs of dark toxicity (Figure 5) [121]. Nanoparticles that respond to changes in pH exhibit favorable activity depending on their size compared to the amount of substance they carry. The process of cancer immunotherapy involves changing nanoparticles with ovalbumin antigens to target murine dendritic cells [172]. Nanoparticles that respond to light showed an improved immune response from CD8+ cells in a living organism [172]. Therefore, a synergistic approach to cancer immunotherapy can be accomplished using photodynamic therapy (PDT). PA’s anticancer efficacy was further enhanced with the addition of cinnamaldehyde, an oxidative-stress-amplifying agent [120]. This combination led to an enhanced generation of ROS through both endogenous and exogenous pathways. The anti-melanoma T-cell immune response was boosted, resulting in reduced tumor growth [120]. A nanoparticle containing acetylxylan–PA was specifically designed for PDT treatment targeting HT-29 colorectal cancer cells [121]. The addition of acetylxylan did not affect the phototoxicity of PA, and there was no significant alteration in the PDT efficacy of both PA and acetylxylan–PA [121]. Paclitaxel was encapsulated in nanoparticles to enhance its photodynamic therapy effect on SK-OV-3 cells [172]. The nanoparticles loaded with paclitaxel generated a higher amount of singlet oxygen compared to free paclitaxel [172]. Although there was not a significant synergistic effect observed, there was a three-fold decrease in cytotoxicity when paclitaxel was used alone to treat cancer cells. Therefore, paclitaxel shows a promising photodynamic therapy effect [172]. PLGA nanoparticles loaded with PA and cisplatin showed a significant enhancement in the antitumor effect against nasopharyngeal carcinoma, resulting in a 4-fold increase in efficacy compared to cisplatin alone in a tumor model resistant to the drug [109]. Several studies showed that the effectiveness of photodynamic therapy for pancreatic cancer can be enhanced through the combined effects of chemotherapy and immunotherapy [173]. Although significant advancements have been made in developing photodynamic therapy for pancreatic cancer, many challenges still exist in moving toward clinical applications. Many uncertainties regarding toxicity and stability are addressed before proceeding to clinical trials. Further research is required to understand the immune response against cancer in the pancreatic environment. Therefore, advancements in enhancing the phototoxicity of pancreatic cancer can be expected based on future outcomes. PSs exhibit promising outcomes in combating multidrug resistance. It is imperative to comprehend the molecular foundation that governs the mechanism of multidrug resistance in order to effectively treat it. In resistant HepG2, the activation of the c-Jun-N-terminal kinases pathway was induced by PPBa/PDT. Notably, the downregulation of P-gp was successfully accomplished, causing no noteworthy harm to the liver or heart of mice with tumors.

### 2.4. Hypocrellin

Natural photoactive quinone pigments (PQPs), extracted and isolated from Hypocrella bambusae, were discovered in China in the early 1980s. Hypocrellin consists of two important pigments such as hypocrellin A and hypocrellin B derivatives. In traditional Chinese medicine, it has been utilized for various skin conditions when exposed to light. The advantages of hypocrellin include low dark toxicity, high singlet oxygen yield, and the generation of hydroxyl and superoxide radicals. The metabolic half-life of hypocrellin is less than one week, making it more suitable for photodynamic therapy compared to porphyrin. However, the aggregation of hypocrellin within the serum leads to a decrease in therapeutic efficacy. These drawbacks must be taken into consideration when developing new agents with an enhanced absorption window [174]. Hypocrellin A was loaded into upconversion nanoparticles for in vitro cell imaging and in vivo MRI and CT imaging along with photodynamic therapy [86,175]. The pi–pi interaction is observed by examining the conjugation between PSs and quantum dots [87]. The sample illuminated by light exhibits favorable phototoxicity as it generates singlet oxygen, which aids in effective bio-imaging. However, self-aggregation remains unresolved in this formulation, leading to a reduced production of reactive oxygen species (ROS) during the photodynamic therapy (PDT) process. To address this, a calcium phosphate nanorod loaded with hypocrellin b, triggered by near-infrared (NIR) light, was proposed to treat Hela cells [89]. Nanorods were synthesized using CaCl_2_, NH_3_⋅H_2_O, and H_3_PO_4_, then coated with dopamine. The dopamine-modified hypercrellin exhibited a significant absorption range between 650 and 800 nm, with an emission peak at 735 nm. This led to an improved uptake efficiency, resulting in a successful reduction in tumor growth. The synthesized nanoplatform also enabled NIR fluorescence imaging-guided PDT, showcasing remarkable results. Kun song et al. developed FA-PEG-PLA micelles containing hypocrellin B, a biodegradable polymer poly(ethylene glycol) (PEG)-poly(lactic acid) (PLA), to achieve active targeting on ovarian cancer [123]. The high encapsulation efficiency in SROV3 FR+ led to a slower drug release compared to the free drug and micelles. A 20-fold increase in drug accumulation was noted when compared to other forms [123].

**Figure 6:**
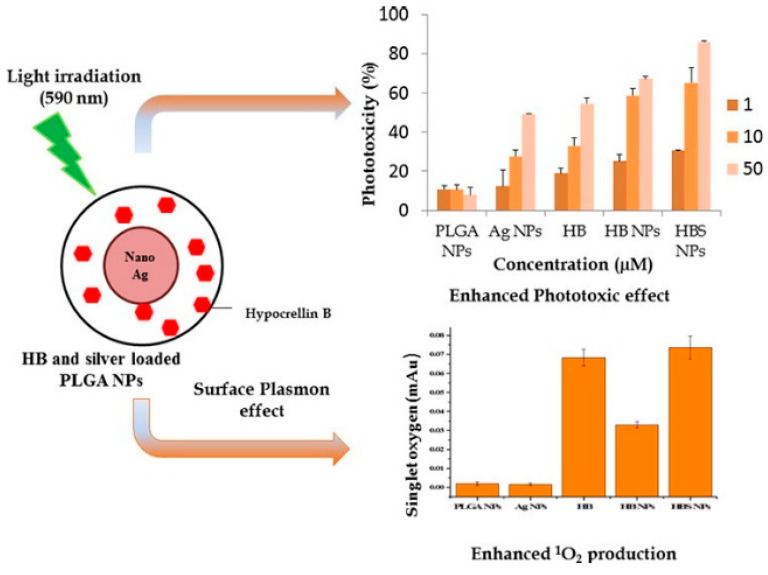
Schematic illustrations of HB and silver nanoparticles loaded with PLGA nanoparticles that were prepared for this study. The focus was on investigating the phototoxicity and singlet oxygen production of HB under light irradiation. To achieve this, polylactide-co-glycolide nanoparticle formulations were developed by incorporating HB and nanosilver enhances the toxicity against cancer cells. Reprinted with permission from Ref. [178]. 2017. Elsevier B.V.

Chuanshan Xu et al. fabricated Irgd peptide-based nanoparticles for fluorescence image-guided photodynamic therapy [106]. Irgd molecules underwent self-assembly to transport hypocrellin in which peptide act as an switch for targeting cancer cells. The Irgd peptide, which specifically targets tumor cells, can reactivate the PS activity [124]. A three-fold increase in penetration depth was observed compared to the control group [124]. The amphiphilic peptide demonstrates excellent penetration depth and enhanced anticancer activity [124]. Tumor-targeted PDT was accomplished with minimal side effects. HA-modified ceramide micelles efficiently encapsulating both a PS and anticancer drug significantly improved the PDT effect in a lung cancer model [110]. Combinational therapy shows a significant anticancer impact on both in vivo and in vitro models. This derivative exhibits strong efficacy compared to the hematoporphyrin derivative PDT. Newly developed micelles are presented as an effective drug delivery system in PDT for lung cancer. Natesan et al. (2017) fabricated hypocrellin B and nanosilver-loaded polylactide-co-glycolide-based nanoparticles using a nonprecipitation technique to enhance singlet oxygen (^1^O_2_) production, resulting in time-dependent phototoxicity against A549 (human adeno lung carcinoma) cells upon exposure to light [110]. Transferrin-modified poly(D,L-Lactide-co-glycolide (PLGA) and carboxymethylchitosan (CMC) nanoparticles are loaded with hypocrellin A [176]. These nanoparticles can enter cells by utilizing the overexpressed transferrin receptor (TFR) through TFR-mediated endocytosis [87]. In this study, A549 cells exhibited elevated levels of reactive oxygen species (ROS) production along with increased apoptosis [176]. A notable 63% tumor inhibition rate was observed, with minimal side effects observed in normal organs [111]. Qin et al. fabricated an inorganic–organic nanocomplex using a folate-modified lipid bilayer spread on PS-entrapped mesoporous silica nanoparticle (MSN)-coated gold nanorods (AuNRs) [111]. Zhang et al. successfully developed biodegradable PLGA nanoparticles coated with a naïve neutrophil membrane, loaded with hypocrellin, for near-infrared fluorescence (NIR FL) imaging and photodynamic therapy (PDT) targeting hepatocellular carcinoma (HCC) [112]. The irradiation of these nanoparticles resulted in the conversion of gold inside the carrier into heat, which generated reactive oxygen species (ROS) to combat cancer [112]. Notably, there were no observed toxic effects on major organs, and the nanoparticles exhibited excellent synergistic photothermal and photodynamic efficiency against tumors. This study highlights the potential of utilizing JUNB/ROS signaling to treat HCC (Figure 6) [112]. The nanoparticle designed to imitate nature exhibits enhanced therapeutic effectiveness and precise targeting toward the tumor site, surpassing challenges posed by blood circulation and immune elimination both in vivo and in vitro. Magnetic mesoporous nanoparticles coated with red blood cell membranes facilitated the delivery of hemoglobin [177]. The nanoparticles were coated with RBCs to enhance their circulation and improve the effectiveness of PDT. By applying a magnetic field against the tumor, ROS were generated [178]. The combination of external magnetic intervention and focused light irradiation successfully eradicated tumor growth [177]. Hb was incorporated into upconversion nanoparticles using Tween 20 [177]. NIR light irradiation triggered the production of ROS from the PS, enhancing imaging and PDT against cancer cells [111]. Multifunctional nanoparticles were created by modifying hypocrellin derivatives with cyclic peptide to combat malignant glioblastoma [90]. These nanoparticles exhibited good photothermal conversion efficiency, increased singlet oxygen production, and in vivo fluorescence imaging [90]. These findings show that the multifunctionalization of nanoparticles holds promise for the diagnosis and treatment of glioblastoma. Various types of nanoparticles were synthesized using PSs to improve photodynamic therapy. Different mechanisms were observed in different cancer cells, possibly because of their unique action mechanisms. Understanding these mechanisms will contribute to the development of more effective clinical applications.

### 2.5. Riboflavin

**Figure 7:**
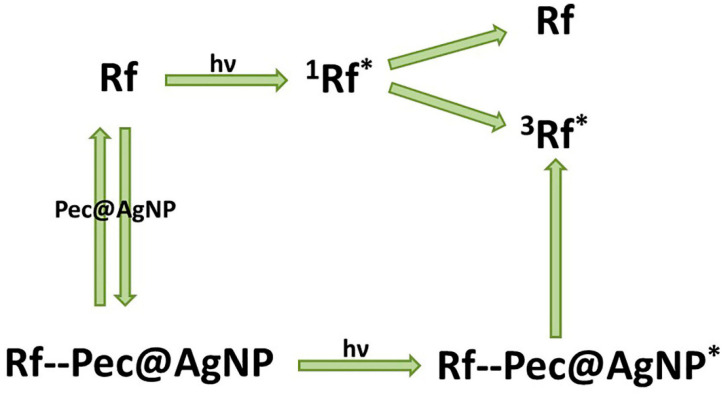
Schematic illustration of effect of Pec@Ag on the generation of the triplet excited state of Rf (^3^Rf*) by the presence of pectin-coated silver nanoparticles (Pec@AgNP) due to the formation of a complex between Rf and Pec@AgNP (Rf-Pec@AgNP). Pec@AgNP* represents the formation of superoxide radicals when irradiated with light. These radical are toxic against cancer cells. Reprinted with permission from Ref. [92] 2018. American Chemical Society.

Riboflavin, a vitamin, can generate an increased amount of reactive oxygen species (ROS) when exposed to UV–blue radiation. These ROS can hinder the growth of cancer cells. Riboflavin is an FDA-approved dietary supplement that exhibits phototoxicity, making it a favorable option for photodynamic therapy [179]. However, the limited penetration of UV–blue light into intricate tissues remains a challenge for its biological applications [88,180,181,182,183,184]. Solubility is also the major concern when PA present in the non-polar solvent reduces its bioavailability, which directly affects its PDT efficiency. The development of PA-based derivatives can be useful in understanding their PS activity, which paves the way for the development of successful PSs in PDT. Nanotechnology also provides a better dimension in the development of PA, retaining its high quantum yield of singlet oxygen generation and non-toxicity [179]. Poly (lactic-co-glycolic acid) nanoparticles, when paired with a riboflavin analog, demonstrate dual effectiveness in targeting triple-negative breast cancer cells [180]. The cellular uptake of these nanoparticles increased six-fold, and they exhibited good cytotoxicity when combined with UV irradiation and loaded with doxorubicin [182]. These synthesized nanoparticles possessed the capability to deliver drugs while also providing a photodynamic effect against TNBC [180]. Photostable derivate RTA was loaded onto mesoporous silica-coated sodium yttrium fluoride: ytterbium/thulium nanoparticles [91]. A core–shell structure was created specifically for phototransducers in the PDT treatment [177]. When exposed to NIR light, the production of ROS was enhanced [180]. In vitro results further confirm that these nanoparticles have the potential to be used as a candidate for PDT. Upconversion nanoparticles aid in activating the NRF through NIR light irradiation. Through in vivo experiments, it was observed that human breast adenocarcinoma cells were selectively and efficiently killed [180]. Pectin-coated silver nanoparticles loaded with fb were used for photodynamic therapy against HELA cells (Figure 7) [92]. Alterations in the DNA and nuclear proteins were detected using synchrotron infrared microscopy. Encouraging photodynamic effects were observed in mammalian cells.

### 2.6. Hypericin

**Figure 8:**
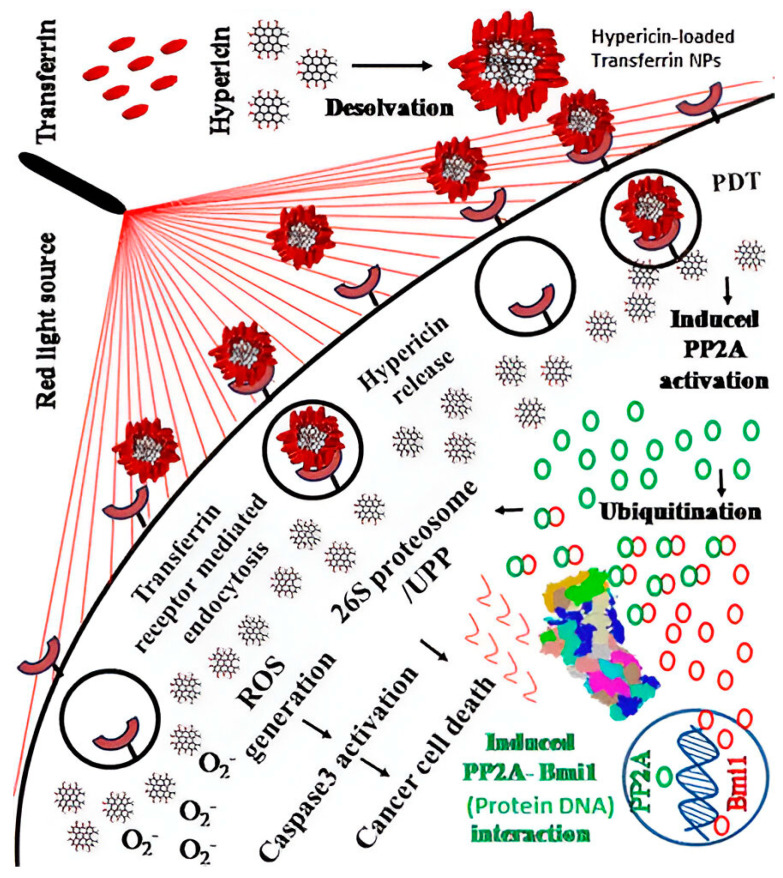
Schematic illustration of hypericin-loaded transferrin nanoparticles and their mechanism of action by inducing PP2A-mediated BMI1 ubiquitination/degradation under light irradiation. “Adapted with permission from Ref. [192] 2020. American Chemical Society”.

Natural polycyclic quinone can be extracted from St John’s Wort (*Hypericum perforatum* L.). This compound exhibits two significant absorption peaks at 545 and 590 nm, making it highly effective in harnessing light energy [105]. The PS can trigger apoptosis and necrosis in different cancer cells via multiple pathways. In the absence of light, hypericin (HP) can hinder the effectiveness of chemotherapeutic medications, thus affecting the overall outcome of cancer treatment [185]. Also, light penetration plays a huge role in the HP PDT effect. Light penetration is limited when treating deep tumor areas, which diminishes PA treatment efficiency [186,187]. The challenge can be overcome by utilizing a range of colloidal carriers as liposomes, emulsions, and microparticles, as discussed earlier [186,187]. In recent years, there has been a growing interest in using nanoparticle-loaded HP for biocompatible drug delivery systems [188,189,190]. For instance, Chen et al. synthesized a theranostic nanoplatform based on gd3+ and hypericin PS through in situ hydrolysis precipitation [191]. The nanoplatform exhibited multifunctional capabilities, including nuclear magnetic resonance (NMR) imaging and anticancer activity. The anticancer activity was achieved by depleting ATP and using the heavy atom effect to enhance photodynamic therapy (PDT) mechanisms. In their study, Damke et al. developed Pluronic ^®^ P123 nanoparticles encapsulating HP to target cervical cancer [125]. The HP nanoparticles induced cell death by inducing cellular oxidative stress, primarily through the type II mechanism of PDT [125]. They inhibited cancer cell migration and invasion by suppressing MMP-2 activity [125]. The application of HP/P123 PDT led to a potent and specific antitumoral impact on cervical cancer cells infected with HPV 16 and HPV 18 [125]. To combat drug resistance in colorectal cancer, transferrin nanoformulations loaded with hypericin were developed (Figure 8) [192]. The activation of PP2A-mediated BMI1 ubiquitination/degradation was detected in the cancer cells [125]. Cell cycle arrest was also observed, along with an increase in ROS production [192]. The effect of nanocapsules on HELA and HEK cells was investigated to understand the behavior of the nanocapsules. When compared to free drugs, the nanocapsules loaded with HP showed elevated ROS production. As a result, cell survival rates of 50–60% and 10–20% were observed in the respective cells [103]. Chitosan-loaded HP improves prolonged circulation and enhanced HP cytotoxicity and improved the uptake efficiency in A549 cancer cells with the help of chitosan. The release of lactate dehydrogenase was elevated in A549 cells, resulting in apoptosis and secondary necrosis because of a 1.6-fold higher production of ROS compared to free HP [193]. Also, Molecular nanogel were developed for the successful delivery of HP and Rose Bengal into HT-29 cells [194]. Nanogel successfully delivers the cargo into the cancer cell for improved PDT efficiency compare to the HP and RB alone respectively.

### 2.7. 5-Aminolevulinic Acid

**Figure 9:**
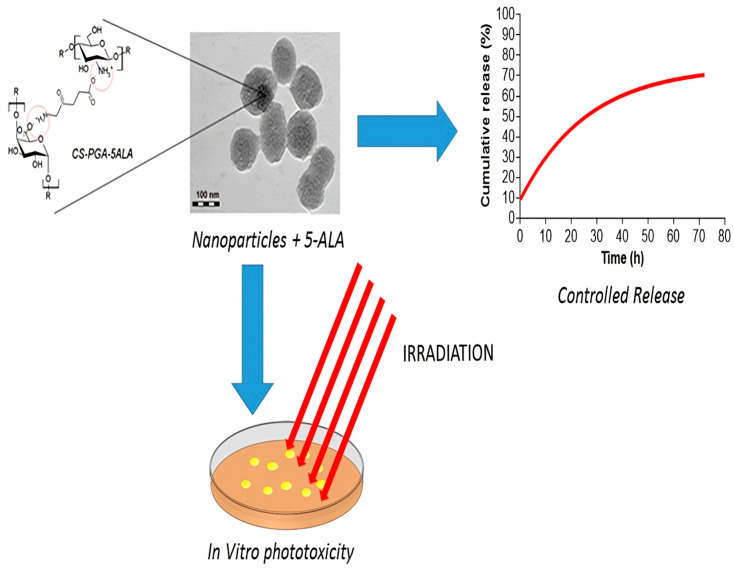
Schematic illustration of the preparation of CS-PGA-5-ALA nanoparticles and their phototoxicity under light illustration. The nanocomplexes were prepared by using chitosan as a polycation and alginic and polygalacturonic acid as polyanions through coacervation. “Reprinted with permission from Ref. [126]. 2017. Elsevier B.V.”.

The FDA has approved 5-aminolevulinic acid as the precursor for PDT [195]. This acid, known as a protoporphyrin IX (PpIX) precursor, has minimal side effects. It enters the cancer cell through peptide transporter 1 and 2 and is subsequently converted into PpIX by mitochondria. However, there are certain limitations to its effectiveness, including inadequate local bioavailability, instability in physiological conditions, and limited diffusion into the cell membrane because of its polarity [94,196]. A mild elevation in liver enzymes, brief skin photosensitivity, nausea, vomiting, and hypotension were observed in treated patients. Various limitations such as poor tissue contrast and spatial resolution, cost, and length of time added to surgery should be considered when developing 5-ALA-based PDT. Several studies have shown that nanocarriers containing 5-ALA might address these limitations. Different nanoparticles were combined with 5-ALA for PDT against a variety of cancer cells. Gold nanoparticles, liposomes, chitosan nanoparticles, mesoporous silica nanoparticles, fullerene, magnetic nanoparticles, quantum dots, upconversion nanoparticles, and bimetallic nanoparticles are just a few examples [80,96,97,98,113,197]. The modification of nanoparticles with antibodies can enhance their uptake compared to bare nanoparticles. Multifunctional agents, dual therapy, and triple therapy have been explored alongside 5-ALA. Sonodynamic therapy, X-ray-induced photodynamic therapy, chemotherapy, photothermal therapy, fluorescence imaging, photoluminescence imaging, and magnetic resonance imaging were also investigated in conjunction with 5-ALA (Figure 9) [80,93,95,126]. Srinivasulu et al. synthesized a 5-ALA-loaded gold nanocluster for overcoming resistance against non-small-cell lung cancer [96]. Image-guided photodynamic therapy can eradicate the 2d spheroids compared to the single compartment [96]. Bismuth nanoparticles have been used as the drug delivery agent for 5-ALA as the PDT against KB cancerous cells [93]. Folic-acid-conjugated bismuth nanoparticles show better efficiency against cancer cells [93,197]. 5-ALA was conjugated with silver nanoparticles which were produced by a bacterial strain called Bacillus licheniformis [95]. The conjugate exhibited increased cytotoxicity against both cell lines in comparison to free 5-ALA, as it generated a significant amount of ROS through PDT [95]. Gold nanoparticles were prepared utilizing *E. coli* and then linked with 5-ALA [96]. The impact of nanoparticles on B16F10 cells and A431 cells was examined for their antitumor properties [95]. The findings indicated a resemblance to the silver–5-ALA conjugate, proposing that the photodynamic therapy (PDT) effect of 5-ALA was attained by triggering protoporphyrin (PpIX) with light exposure [96]. The successful implementation of combined chemo-photodynamic therapy (CPDT) was conducted through intratracheal administration on A549 cells (adenocarcinoma human alveolar basal epithelial cells) and primary lung cancer rats [97]. This was achieved by preparing gefitinib PLGA nanoparticles with 5-ALA. Noteworthy lung cancer markers such as CD31, VEGF, NF-κB p65, and Bcl-2 were notably decreased because of the synergistic effect of CPDT compared to single therapy [98]. The dual therapy effectively regulated inflammation by reducing TNF-α signaling, which another significant outcome was observed through the impact of nanoparticles [98]. An ALA prodrug nanocarrier was created by utilizing a hydrazine linkage to connect gold nanoparticles with MMP-2 cell-penetrating peptides [198]. These peptides can activate within the tumor microenvironment, allowing the nanocarrier to remain in circulation and target the tumor site for an extended period [113]. The activation of PpIX generation leads to photodynamic tumor inhibition, which is facilitated by the development of a tumor microenvironment-sensitive prodrug nanocarrier [97]. To study the effect of photodynamic therapy (PDT) on human chronic myeloid leukemia K562 cells, gold-conjugated 5-ALA was created [96]. The PDT mechanism involves the generation of singlet oxygenation through light irradiation by the gold nanoparticles [98]. The results of the study also show that gold-conjugated 5-ALA effectively kills cancer cells by generating singlet oxygen through the gold nanoparticles themselves [98].

### 2.8. Pheophytin

**Figure 10:**
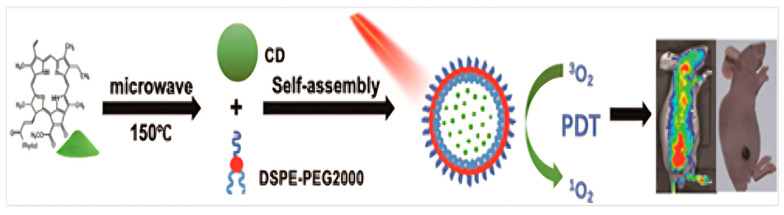
Schematic illustration of CD assembly derived from pheophytins for FL imaging and synergistic PDT of cancer. Pheophytin is a type of Mg-free chlorophyll derivative used for the preparation of CDs by using a microwave method and self-assembled with DSPE-mPEG2000 for efficient ^1^O_2_ generation and efficient fluorescence (FL) imaging. Reprinted with permission from Ref. [99]. 2019. Wiley.

Pheophytin, a chlorophyll derivative devoid of magnesium, is naturally present in plants for the process of photosynthesis. Its structure bears a resemblance to the porphyrin structure. Pheophytin holds potential as a photoactive compound. However, its main limitations lie in its instability and hydrophobic nature, rendering it unsuitable for certain applications [198]. A carrier made of ROS-degradable Thioketal-cross-linked polyethyleneimine was utilized to deliver pheophytin A and the p53 gene [199]. ROS generated by far-red-light irradiation aid in the release of the p53 gene into the cancer cells [119]. An enhancement accompanied the enhanced anticancer activity in the stability of pheophytin A. Using the surfactant stripping method, micelles were created to facilitate improved photodynamic therapy. The ss-pheophytin micelles successfully achieved a high singlet oxygen quantum yield, prolonged circulation within the bloodstream, and targeted accumulation at the tumor site [119]. Carbon dots were used to load the PS to improve the hydrophobicity. Dspe-mpeg 2000 was self-assembled on the surface of the carbon dot–pheophytin nanoparticles (Figure 10) [99,100]. Exceptionally, PTT and PDT were observed through fluorescence imaging, showcasing high bioavailability. Lipid–PEG/pheophytin carbon dots enhanced the photo effect via the protein corona effect. These structures pave the way for novel applications in targeted drug delivery. Multimodal imaging and cancer-targeting nanoplatforms play a crucial role in eradicating metastatic breast cancer.

### 2.9. Bacteriochlorin

**Figure 11:**
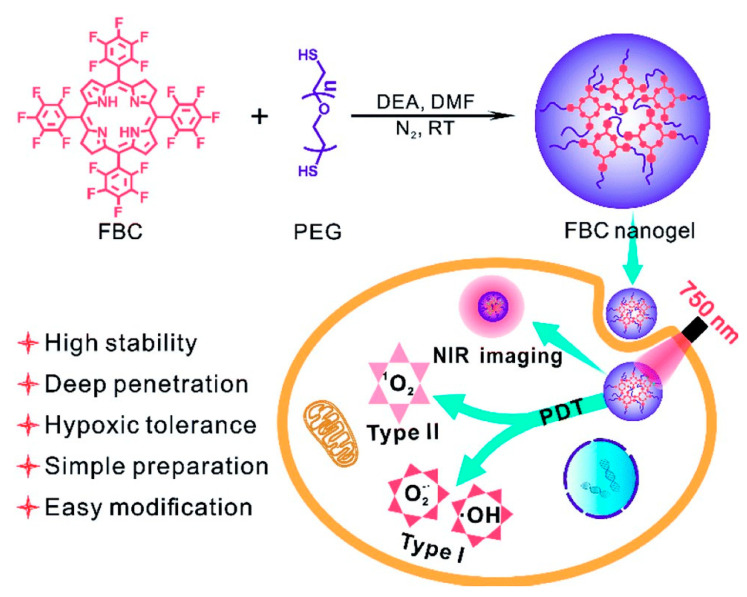
Schematic illustration of the synthesis of FBC nanogel and the mechanism of the PDT performance of the FBC nanogel. A bacteriochlorin analogue, tetra fluorophenyl bacteriochlorin, was developed by the one-step reduction of tetra fluorophenyl porphyrin (TFPP). A biocompatible FBC nanogel could be directly formed by blending FBC with SH–PEG–SH. “Adapted with permission from Ref. [127]”.

Bacteriochlorin exhibits strong absorption in the near-infrared (NIR) range of 700–900 nm. Despite their ability to penetrate deep into tissues, most metal derivatives of bacteriochlorophylls proved to be unstable [200]. Ongoing research is being conducted to develop stable versions of bacteriochlorin derivatives that have improved resistance to light degradation. Nanoparticles containing bacteriochlorin have shown promise as an effective method for generating reactive oxygen species in photodynamic therapy for cancer treatment. Recently, scientists have been investigating the use of magnetic nanoparticles loaded with bacteriochlorin to enhance its availability in the body and enable MRI-guided photodynamic therapy [101]. By loading bacteriochlorin onto MNP-HSA-PEG, it becomes possible to track its movement using MRI, allowing for personalized monitoring of how the photosensitizer behaves at the tumor site [101]. This approach also offers advantages, such as strong T2 contrast properties and a high ratio of effectiveness in the absence of light compared to light [101]. Also, magnetic nanoparticles were coated with human albumin serum for improved noninvasive MRI imaging [101]. It was evidenced that the formulation has the potential to serve as a superior tumor-targeting agent with enhanced efficacy. A nanogel was created by incorporating a bacteriochlorin analog with SH–PEG–SH (Figure 11) [127]. This nanogel exhibited remarkable photodynamic efficiency in both in vivo and in vitro settings. Additionally, Zr-TBB metal–organic frameworks were employed to immobilize bacteriochlorin to treat breast and colon cancer [132]. Upon light irradiation, ROS, including singlet oxygen, superoxide anions, hydrogen peroxide, and hydroxyl radicals, were generated in the mouse model with 40 and 60% efficiency.

### 2.10. Berberine

Berberine, an isoquinoline alkaloid derived from different Chinese herbal remedies commonly used in traditional Chinese medicine, serves as an antibacterial, immunostimulant, anticancer, and antineoplastic agent [201,202]. Various pathways and molecular targets in cancer cells are targeted to start apoptosis. The expression of genes that inhibit apoptosis is reduced, while the expression of genes that promote apoptosis is increased. Specific effects on cell migration and activity related to caspase 3, autophagy, and the aging of antineoplastic cells were observed. A photoactive compound can generate toxic ROS, which enables it to exert a photodynamic effect against cancer cells [203,204]. Various reports show that berberine can be used as the PS in PDT [205,206,207]. The primary limitation of most Chinese medicine lies in its bioavailability. A recent investigation delved into the photodynamic impact of berberine, specifically the nanoemulsion, as a potential treatment for cervical carcinoma [133]. The phototoxicity of a berberine nanoemulsion against cervical carcinoma cells and human keratinocyte cells was evaluated statically [133]. Under light excitation, the nanoemulsion induced autophagy in cancer cells, with no significant caspase activity observed in either cell line [133]. The results suggest berberine incorporated in nanoform could serve as a more effective photoagent for targeting various tumors [133]. Berberine nanoparticles were developed by encapsulating them in PLGA, stabilized with chitosan oleate and further coated with folic acid for cancer targeting through the overexpression of folate [208]. This mitochondria-targeting agent induced toxicity in the T98G GBM-established cell line [209]. A berberine dimer nanocomposite incorporated with gold nanostars was used as a PDT and PTT agent against breast cancer cells. HA-change gold stars could target CD44 receptors overexpressed on the tumor. Gold stars not only facilitated berberine for PDT but also acted as the PTT agent. Therefore, the prepared nanocomposite shows promise as an in vitro agent and exhibits anti-breast-cancer activity in vivo through synergistic PDT and PTT. However, further research is needed in the future for berberine photodynamic therapy.

## 3. Strategies to Address Challenges in PDT with Natural PSs against Resistance

In recent years, the realm of nanotechnology and nanoscience has experienced notable progress, resulting in the emergence of a broad spectrum of nanomaterials, each with diverse structures, forms, and dimensions, accessible to researchers [210]. Many investigations show that nanoparticles can improve the dispersal of natural photosensitizers, shield them from deterioration, regulate their distribution within the body, and prolong their circulation in the blood [211]. Nanomaterials can further provide a controlled release mechanism, enhance cellular absorption, and target cancer cells more precisely, minimizing adverse effects and systemic toxicity [212,213]. Combinations of various anticancer agents with nanoparticles have shown the potential to change the phototoxic impacts of photosensitizers, leading to a synergistic or beneficial outcome [75,214]. These features present optimism for the creation of novel, innovative photodynamic therapy strategies that might replace existing conventional treatments. It is crucial to acknowledge that only a restricted variety of nanoparticle compositions for photodynamic therapy have advanced to human clinical trials [215]. This poses a considerable obstacle that must be overcome for the effective clinical implementation of these nanosystems [216]. The main cause of the failure of these ps-loaded nanocarriers is the tumor microenvironment. Therefore, by adding extra molecules like receptors and aptamers, or by changing the interaction with specific cell membranes on the nanoparticles’ outer layer, the cancer-targeting ability of the nanocarrier can be further enhanced. Also, developing novel nanocarriers based on the tumor environment will be the innovative strategy to overcome these limitations for natural PS-loaded nanoparticles [217,218,219,220,221]. nMOFs have provided a novel nanotechnology platform to load various PSs by incorporation into bridging ligands or entrapment in the pores/channels, resulting in the improved stability and pharmacokinetic behaviors of natural PSs [222]. Pu Zhang and team developed nanoparticle loaded with Zn(II)-PPIX and UiO-66 metal–organic frameworks for the purpose of imaging cancer cells guided by miRNA and for the simultaneous photodynamic therapy of cancerous cells. Initially, the Zn(II)-PPIX-loaded NMOFs had two hairpins [223]. The hairpins H_i_/H_j_ were engineered for inclusion in the stem region of H_i_, the miRNA recognition sequence, and in their partially locked stem domains’ caged G-quadruplex units. The imaging and PDT of MDA-MB-231 breast cancer cells and of OVCAR-3 ovarian cancer cells were achieved due to the production of ROS with the help of biomarkers miRNA-21 and miRNA-221. This nanoplatform was a successive outcome because of the presence of overexpressed miRNA in the cancer cells. Also, lu et al. developed a chlorin-based nanoscale metal–organic framework for the photodynamic therapy of colon cancers [224]. Wan et al. developed a novel nanoscale MOF (NMOF) for the delivery of DHA cargo from the Fe_3_O (OOC) 6 metal cluster and 4,4,4,4- (Porphine-5,10,15,20-tetrayl) tetrakis (benzoic acid) (TCPP). An Fe-TCPP NMOF was coated with CaCO_3_ to avoid the DHA leakage and phototoxicity of TCPP during transportation in the bloodstream. Triple synergistic therapy methods were achieved because of the tumor microenvironment. The high concentration of intracellular GSH reduced the Fe^3+^ of the NMOF to Fe^2+^ [225].

Lan and their team created a unique magnetic metal–organic framework (MOF) to address low oxygen in tumor treatment during photodynamic therapy (PDT) and to enhance the effectiveness of cancer immunotherapy. They designed Fe-TBP by assembling Fe_3_O clusters and linking them with a 5,10,15,20-tetra (p-benzoate)porphyrin (TBP) ligand [226]. This structure, when exposed to low-oxygen environments, initiated a series of reactions leading to the decomposition of intracellular hydrogen peroxide (H_2_O_2_) by Fe_3_O clusters, which then produced oxygen (O_2_) through a Fenton-like reaction. The resulting O_2_ was further transformed into cytotoxic singlet oxygen (^1^O_2_) by photo-excited porphyrins. Using Fe-TBP in PDT resulted in a systemic antitumor effect, boosting the immune response against cancer through an improved alpha-PD-L1 immune checkpoint blockade (ICB), enhancing systemic antitumor immunity. DNA aptamers, capable of precisely attaching to proteins that are overexpressed in tumors, are widely used to create nanocarriers that can specifically target tumors [212,227,228]. Many sequences of DNA aptamers have been discovered and integrated into these nanocarriers to enhance their ability to target, increasing the effectiveness of tumor photodynamic therapy [229,230]. Sun et al. developed DNA aptamer-conjugated SPION for simultaneous targeting and the integration of chemotherapy and photodynamic therapy [231]. Polydimethylsilyl-Apt-S8@SPION nanocarriers showed promising stability in both serum and in the presence of nuclease DNase I. These nanocarriers demonstrated significant effectiveness in killing A549 and C26 cells, which express an excessive number of nucleoli, a protein often found in cancer cells. Using cell membrane biomimetic technology, these limitations in the photodynamic therapy (PDT) process were overcome. Various cell membrane types have been employed to conceal phototherapeutic agents, including those from red blood cells (RBCs), platelets (PLTs), macrophages, and cancer cells (CCMs). A mixture of cell membranes and engineered cell membranes has been incorporated into nanoparticles to enhance the effectiveness of photodynamic therapy [232,233]. Chen and colleagues created TCPP integrated into NK-NPs to produce a response against cancer [234]. The NK cell membranes allowed NK-NPs to identify and attack tumors, leading to the activation of M1 macrophages, which then turned into M1 macrophage polarization to produce immunotherapy treatment at the cell membrane level. By combining receptor and aptamer molecules or adding certain cell membrane attachments, it is possible to increase the formation of hybrid entities within cancer cells and simultaneously decrease their presence in normal tissues, thereby improving the success of photoacoustic therapy (PDT) and reducing its side effects.

**Figure 12:**
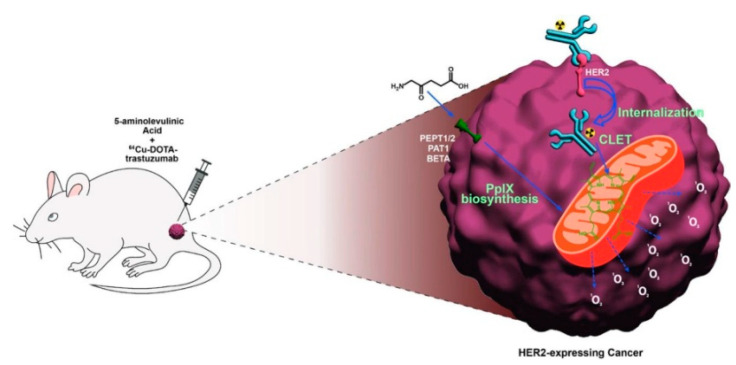
Schematic illustration of the administration of 5-aminolevulinic acid and ^64^Cu-DOTA-trastuzumab based nanocarrier into HER2-overexpressing cancer cells. The nanocarrier generats ^1^O_2_ via Cerenkov luminescence energy transfer (CLET) results in cancer cell death. Reprinted with permission from Ref. [239]. 2019. Elsevier B.V.

Traditional light sources have severe limitations, such as a lack of penetration depth inside the tumor region. Such a problem can be overcome by a technique called self-exciting PDT, which uses radioactive energy to excite PSs [235,236]. Hu and colleagues developed a comprehensive PDT nanosystem (LHHP) by encasing both luminol and hemoglobin within a hierarchically porous metal–organic framework (MOF) for effective light-induced regeneration therapy and self-sufficiency in oxygenation. This assembly featured tetrakis(4-carboxyphenyl)porphyrin (TCPP) as the organic linker, which was evenly distributed within the hierarchically porous porphyrinic MOF (HP-PCN-224) to avoid clustering, and the porous nature of HP-PCN-224 facilitated the binding of hemoglobin to it, thus aiding in the delivery of oxygen [237]. Luminol would react with the overproduced hydrogen peroxide in the tumor area, resulting in the emission of blue chemiluminescence. TCPP captured light energy through a CRET process to produce luminescence, and this light energy was subsequently transferred to the oxygen attached to hemoglobin to form reactive oxygen species (ROS). Following the injection of the LHHP nanocomposite, it was observed to significantly inhibit the growth of tumors beneath the skin in the subcutaneous 4T1 tumor model. This research marked a significant milestone in the field of PDT to treat cancers located deep within the body, using a singular PDT nanosystem that could provide both self-luminescence and self-sufficiency in oxygen supply [238]. Jo and her team developed a sophisticated and successful photo-activated therapy technique using Cerenkov light in PDT, which is based on the unique properties of 5-aminolevulinic acid (5-ALA) and ^64^Cu-DOTA-trastuzumab (Figure 12) [239]. This combination allows for the precise delivery of charged particles to the cancerous area by targeting the ^64^Cu-DOTA-trastuzumab, while the buildup of PpIX at the site facilitates the absorption of Cerenkov light emitted by ^64^Cu within the tumor, leading to the production of cytotoxic oxygen molecules, ^1^O_2_. The researchers created a tumor model under the skin of NCI-N87 cancer cells in nude mice that express high levels of HER2. They showed that the use of this two-targeted approach for the tumor, namely 5-ALA and ^64^Cu-DOTA-trastuzumab, effectively reduced the tumor’s size with no noticeable adverse reactions.

Collectively, all the current strategies have opened a gateway for the progress of natural PSs in PDT. Nanotechnology integrating natural PSs with various strategies has not been successful in the clinical PDT treatment of malignant tumors. However, the research community has been attempting to break through this challenge and will develop the maximum translation significance for natural PSs.

## 4. Conclusions

In this review, we summarized the recent advances in natural PSs in PDT. Given their unique advantages, natural PSs have developed their efficiency against the tumor environment. In recent years, various studies have shown that natural PSs can combat the resistance created by cancer. Natural PSs have a lot of advantages, but they cannot be used as is because of the circumstances created during the treatment. Various new natural PSs have been identified and their photostability and PDT efficicancy can be improved with the help of various nanocarrier. Natural PS derivatives with NIR-II light irradiation can make the in-depth penetration of light across the tumor possible. Also, two-photon treatment, along with PDT, can be a substitute for deep light penetration by improving its efficiency. Various nanocarriers loaded with natural PSs look promising in cancer research. Though MDR is not an easy phenomenon to tackle with a single therapy, multiple therapies can achieve the mark with some special features. To advance the field of nanomedicine, it is crucial to comprehend how natural based PS combact resistance in cancer. Clear information about the tumor environment for early diagnosis, clinical treatment, and postoperative intervention can be studied with the help of imaging. Fluorescence, photoacoustic, and magnetic resonance imaging can be performed along with PDT to understand the response developed during the treatment. Also, combining PDT with other therapies like chemotherapy, radiotherapy, sonodynamic therapy, and immune therapy will have a better therapeutic outcome compared with monotherapies. All-in-one nanoparticles can be the better option for cancer theranostics. Finally, this review will enable us to come up with suitable treatment modalities with minimized side effects. We hope this review will help in understanding the effect of natural PSs in PDT against cancer and its resistance throughout treatment.

## Figures and Tables

**Table 1:** List of Natural Photosensitizers loaded with different types of nanocarriers in PDT for treating cancer.

Nanomaterial Classification	Nanomaterial	Natural PS	Therapeutic Ability	Reference
Inorganic NPs	LDH	Curcumin	PDT	[76,77]
Inorganic NPs	Gold NPs	Curcumin, 5-ALA	PDT	[78,79,80]
Inorganic NPs	Silica	Curcumin	PDT	[81,82]
Inorganic NPs	MoS2	Curcumin, ICG	PDT, PTT	[83]
Inorganic NPs	Ga	Curcumin	PDT	[84]
Inorganic NPs	Sn	Porphyrin	PDT	[85]
Inorganic NPs	Up conversion NPs	Hypocrellin b Hypocrellin a Riboflavin	PDT, MRI, FL, UCL Imaging	[86,87,88]
Inorganic NPs	Calcium phosphate	Hypocrellin b	PDT, FL IMAGING	[89]
Inorganic NPs	MSN	Riboflavin, Hypocrellin b, Curcumin	PDT	[90,91]
Inorganic NPs	Bismuth NPS	5-ALA, Riboflavin	PDT	[92,93]
Inorganic NPs	Silver NPs	5-ALA, Riboflavin	PDT	[94,95]
Inorganic NPs	Gold nanoclusters	5-ALA	PDT	[96,97,98]
Inorganic NPs	Carbon- Dots	Pheophytin	PDT	[99,100]
Inorganic NPs	Magnetic NPs	Bacteriochlorine	PDT, MRI	[101]
Organic NPs	Lipid NPs	Curcumin, Hypericin	PDT	[102,103]
Organic NPs	Liposomes	porphyrin	PDT	[104]
Organic NPs	Micelles	Pheophorbide a, Hypericin	PDT	[85,105]
Organic NPs	Irgd peptide	Hypocrellin b	PDT	[106]
Polymers	PLGA	5-ALA, Riboflavin, Hypocrellin b, Curcumin, Pheophorbide a	PDT, Chemotherapy FL	[107,108,109,110,111,112,113]
Polymers	Chitosan	curcumin	PDT, FL	[114]
Polymers	Arginine complex	porphyrin	PDT	[115]
Polymers	Alginate	Pheophorbide a	PDT, Chemotherapy, Immunotherapy	[116]
Polymers	Pluronic F127	Surfactant-stripped Pheophytin, Pa	PDT, chemotherapy	[117]
Polymers	PEI	Pheophorbide a, Pheophytin	PDT	[118,119]
Polymers	polygalactose-co-polycinnamaldehyde polyprodrug	Pheophorbide a	PDT, Immunotherapy	[120]
Polymers	Xylan	Pheophorbide a	PDT	[121]
Polymers	PEG	Pheophorbide a	PDT	[122]
Polymers	PEG-PLA	Hypocrellin b	PDT	[123]
Polymers	hyaluronic acid–ceramide nanoparticles	Hypocrellin b	PDT, CD	[124]
Polymers	P123 nanomicelles	HYP	PDT	[125]
Polymers	Polysaccharides based nanocomplexes	5-ALA	PDT	[126]
Polymers	SH–PEG–SH	tetrafluorophenyl porphyrin	PDT	[127]
Hybrid NPs	Metal organic framework	Porphyrin, Bacteriochlorin	PDT, PTT, Chemotherapy, Immunotherapy	[128,129,130,131,132]
Hybrid NPs	Polysilsesquioxane nanoparticles	5-ALA	PDT	[80]
	Transferrin Nanoparticles	Hypericin	PDT, Chemodynamic Therapy	[103]
	Nanoemulsion	berberine	PDT	[133]

**Table 2:** Structures of various natural photosensitizers used in photodynamic therapy studies.

Photosensitizers	Structure	Wavelength
Curcumin	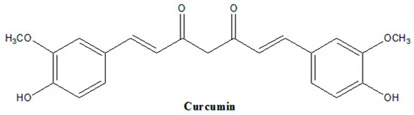	420–480 nm
Bacteriochlorin	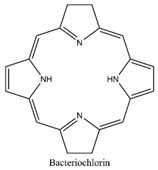	700–900 nm
Berberine	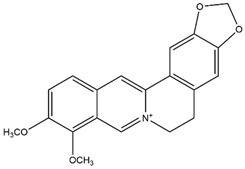	250–350 nm
5-aminolevulinic acid	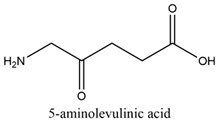	405 and 635 nm
Hypericin	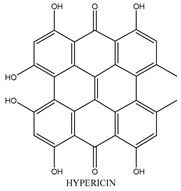	590 nm
Riboflavin	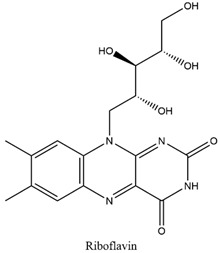	365–445 nm
Pheophorbide a	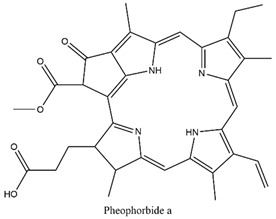	670 nm
Porphyrin	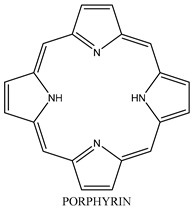	600–900 nm
Pheophytin	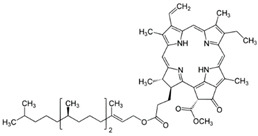	400–700 nm

## Abbreviations

MDR	Multidrug resistance
PSs	Photosensitizers
PDT	Photodynamic therapy
PTT	Photothermal therapy
MoS_2_	Molybdenum disulfide
ICG	Indocyanine green
ROS	Reactive oxygen species
CUR	Curcumin
ABC	ATP-binding cassette
NIR	Near-infrared light
MRI	Magnetic resonance imaging
HY	Hypericin
UCL	Upconversion luminescence imaging
FL	Fluorescence imaging
PAT	Photoacoustic imaging
PET	Positron emission tomography
PEG	Polyethylene glycol
Pa	Pheophorbide a
PpIX	Porphyrin
BER	Berberine
Rf	Riboflavin
SLNs	Solid lipid nanoparticles
5-ALA	5-Aminolevulinic acid
MNPs	Magnetic nanoparticles
HAS	Human serum albumin
PLGA	Poly(lactic-co-glycolic acid)
MSNs	Mesoporous silica nanoparticles
CMC	Carboxymethylchitosan
FA	Folic acid
HA	Hyaluronic acid
MOF	Metal–organic framework
MB	Methylene blue
DNA	Deoxyribonucleic acid
Bcl-2	B-cell lymphoma-2
Bcl-xl	B-cell lymphoma-extra-large
NF-κB	Nuclear factor kappa-light-chain-enhancer of activated B cells
ER	Endoplasmic reticulum
EPR	Enhanced permeability and retention effect
